# Supplementary material for: Child Growth Curves in High-Altitude Ladakh: Results from a Cohort Study
Source: Int J Environ Res Public Health. 2020 May 22;17(10):3652. doi: 10.3390/ijerph17103652 (PMC7277569; doi:10.3390/ijerph17103652)
Supplement: Supplementary file 1 [file ijerph-17-03652-s001.pdf]

Table S1. The means of Z scores of body weight, height, and body mass index during 2012–2018.

	Descriptive Analysis, *Z* Score Mean (SE)			Analysis Using GEE, *Z* Score Mean (SE)		
	**BW**	**BH**	**BMI**	**BW**	**BH**	**BMI**
**2012**	−1.71 (0.08)	−1.73 (0.08)	−0.99 (0.07)	−1.47 (0.07)	−1.44 (0.07)	−0.85 (0.06)
**2013**	−1.43 (0.09)	−1.61 (0.08)	−0.84 (0.06)	−1.31 (0.06)	−1.37 (0.07)	−0.71 (0.05)
**2014**	−1.22 (0.09)	−1.37 (0.08)	−0.91 (0.06)	−1.24 (0.07)	−1.20 (0.07)	−0.79 (0.05)
**2015**	−1.01 (0.09)	−1.32 (0.07)	−0.76 (0.05)	−1.09 (0.07)	−1.18 (0.07)	−0.67 (0.05)
**2016**	−0.89 (0.08)	−1.27 (0.07)	−0.64 (0.05)	−0.90 (0.06)	−1.16 (0.06)	−0.54 (0.05)
**2017**	−0.76 (0.08)	−1.14 (0.07)	−0.66 (0.05)	−0.86 (0.06)	−1.10 (0.06)	−0.58 (0.05)
**2018**	−0.57 (0.08)	−0.92 (0.07)	−0.66 (0.05)	−0.74 (0.07)	−0.92 (0.06)	−0.63 (0.05)

BW: body weight, BH: body height, BMI: body mass index, SE: standard error, GEE: generalized estimating equation.

Table S2. Annual growth rates of body height stratified by age and gender.

**Age (years)**		**BH Change (cm/year)**			
**From**	**To**	**Boys**		**Girls**	
		**Mean (SE)**		**Mean (SE)**	
4	5	5.97	(0.37)	6.03	(0.69)
5	6	5.99	(0.21)	6.39	(0.35)
6	7	5.75	(0.26)	6.07	(0.24)
7	8	5.50	(0.36)	5.76	(0.23)
8	9	4.67	(0.37)	6.01	(0.20)
9	10	5.32	(0.16)	6.34	(0.23)
10	11	5.96	(0.27)	6.52 *	(0.21)
11	12	5.50	(0.25)	6.51 *	(0.24)
12	13	6.75	(0.41)	5.77	(0.27)
13	14	7.07 *	(0.40)	4.36	(0.30)
14	15	7.02 *	(0.56)	2.77	(0.31)
15	16	4.25	(0.89)	1.95	(0.30)
16	17	4.54	(1.28)	1.14	(0.32)
17	18	3.39	(3.68)	2.25	(0.81)

* the 2 years with the maximum growth rates for boys and girls. BH: body weight, SE: standard error.
